# An Implementation Science Study of a Heat-Producing Wrap to Complement KMC in Rwanda

**DOI:** 10.5334/aogh.4430

**Published:** 2024-07-29

**Authors:** Florent Rutagarama, Cyiza Francois Regis, Christian Umuhoza, Lisine Tuyisenge, Cynthia Grace Mfuranziza, Pacifique Hagenimana, Micaela Matteo Smith, Henry A. Feldman, Anne R. Hansen

**Affiliations:** 1 Rwanda Paediatric Association, University of Rwanda, Rwanda Military Hospital, Rwanda; 2 Rwanda Biomedical Center, Ministry of Health, Rwanda; 3 Rwanda Paediatric Association, University of Rwanda, University Teaching Hospital of Kigali, Rwanda; 4 Rwanda Paediatric Association, University Teaching Hospital of Kigali, Rwanda; 5 Rwanda Paediatric Association, Rwanda; 6 Boston Children’s Hospital, Boston MA

**Keywords:** Neonatal hypothermia, implementation science, newborn, kangaroo mother care (KMC)

## Abstract

*Background:* Neonatal hypothermia is a major cause of preventable morbidity and mortality, especially among the world’s poorest newborns. A heat-producing wrap is necessary when kangaroo mother care (KMC) is insufficient or unavailable, yet there is little published research on such wraps. The Dream Warmer is a wrap designed to complement KMC and has been extensively studied in formal research settings but not in real-world conditions.

*Objectives:* We used implementation science methodology to understand the safety, effectiveness, and functionality of the Dream Warmer (hereafter, “Warmer”); its effect on clinical workflows; its interaction with other aspects of care such as KMC; and the Warmer’s reception by healthcare providers (HCPs) and parents.

*Methods:* We conducted a prospective, interventional, one-arm, open-label, mixed-methods study in 6 district hospitals and 84 associated health centers in rural Rwanda. Our intervention was the provision of the Warmer and an educational curriculum on thermoregulation. We compared pre and post intervention data using medical records, audits, and surveys.

*Findings:* The Warmer raised no safety concerns. It was used correctly in the vast majority of cases. The mean admission temperature rose from slightly hypothermic (36.41 °C) pre, to euthermic (36.53 °C) post intervention (*p* = 0.002). Patients achieved a temperature ≥36.5 °C in 86% of uses. In 1% of audits, patients were hyperthermic (37.6–37.9 °C). Both HCPs and parents reported a generally positive experience with the Warmer. HCPs were challenged to prepare it in advance of need.

*Conclusions:* The Warmer functions similarly well in research and real-world conditions. Ongoing education directed toward both HCPs and parents is critical to ensuring the provision of a continuous heat chain. Engaging families in thermoregulation could ease the burden of overtaxed HCPs and improve the skill set of parents. Hypothermia is a preventable condition that must be addressed to optimize neonatal survival and outcome.

## Background

**Figure 1:**
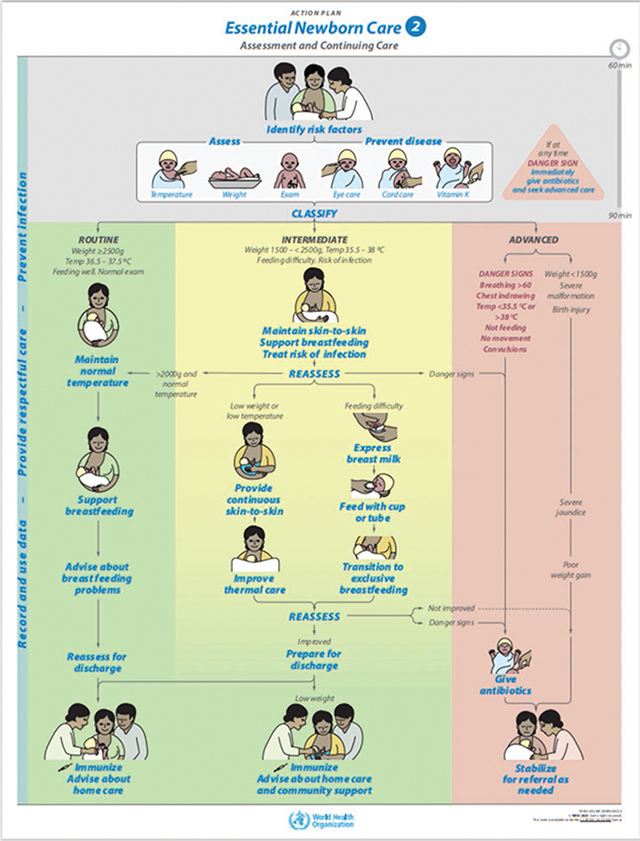
Essential Newborn Care 2 Assessment and Continuing Care.

Neonatal hypothermia is widely recognized to be one of the major causes of preventable morbidity and mortality, especially among the world’s poorest newborns. It is estimated to contribute to 40% [1] of the 2.3 million neonatal deaths annually [2], almost exclusively in low- and middle-income countries. Those who survive suffer from stunted growth and brain development as calories are shunted toward thermoregulation [3]. Provision of warmth is an essential element of the most basic care of small and sick newborns, yet it is frequently lacking; equipment such as incubators and radiant warmers are cost-prohibitive, difficult to use and maintain, and reliant on a constant electrical supply. In low-resource settings, the World Health Organization (WHO) recommends KMC for stable newborns at risk of hypothermia [4]. While KMC is a highly effective, low-cost intervention, it does not always provide sufficient heat, and maternal pain and fatigue represent significant barriers [5]. In these circumstances, the WHO’s Essential Newborn Care (ENC) 2 guideline recommends “improved thermal care” with the addition of a heat-producing wrap (Figure 1) [6]. Though use of such a product meets a widely recognized need, there are limited published data on the safety, effectiveness, and usability of available options, or of their effect on HCPs’ and parents’ experience.

**Figure 2:**
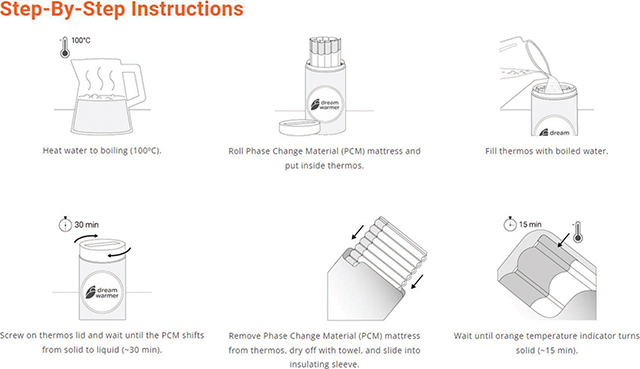

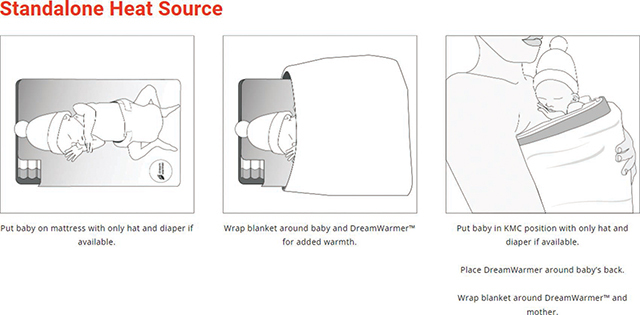
Low Literacy Instructions for Preparation and Use of the Dream Warmer (DreamWarmer™ – Global Newborn Solutions).

The Dream Warmer is a heat-producing wrap based on the concept of a skin-temperature heating pad, incorporating an enclosed wax that, once melted by boiled water, remains at 37 °C for approximately 6 hours. It includes a 40 °C temperature indicator that clarifies adequate cooling to initiate use. As with skin-to-skin care, the infant is then laid naked directly on the Warmer, either additive to KMC or as a standalone heat source (Figure 2). To date, three clinical trials of the Dream Warmer have been performed in Rwanda: two pilot studies [7,8] and one large-scale Cluster Randomized Stepped Wedge Trial [9]. The aggregate results of these studies, spanning 1074 uses, show that the Warmer raised no safety concerns except a 9% rate of mild hyperthermia, compared to a 12% rate in patients who did not use the Warmer [9], reflective of the generally underdeveloped ability of premature newborns to regulate their body temperature. The Warmer was highly effective with 92% of patients either becoming euthermic (when used for hypothermic patients) or remaining euthermic (when used for low-birthweight patients when KMC was unavailable). It is now professionally manufactured and commercially available. The United States Food and Drug Administration determined it to be a “product” and not a “device,” and therefore outside of their purview. It has been approved as a class A device by the Rwanda FDA and cleared by TUV, a third-party safety verification operation. Thus, the Warmer is a low-cost, affordable, easy-to-use heat-producing wrap to complement KMC that can reduce rates of neonatal hypothermia in low-resource settings.

While this heat-producing wrap has now been adequately studied in a controlled research context, it remains necessary to characterize the human and behavioral factors that influence its implementation, uptake, and use in real-world conditions. This implementation science study was designed to gain a deep understanding of the Warmer’s safety, effectiveness, and functionality; its effect on clinical workflows; its interaction with other aspects of care such as KMC; and its reception by HCPs and parents.

## Methods

**Figure 3:**
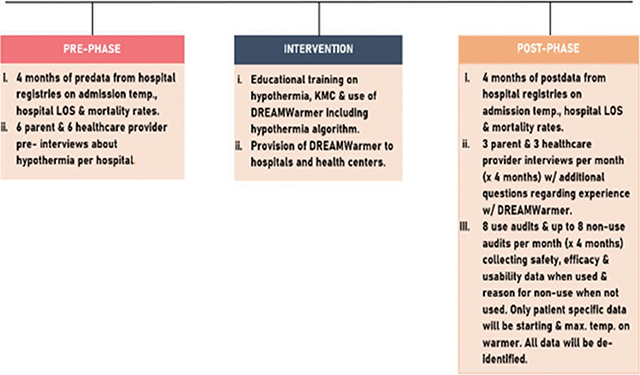
Study Design.

We conducted a prospective, interventional, one-arm, open-label, mixed-methods study in district hospitals and associated health centers in rural Rwanda (Figure 3). Our intervention started with a 30-minute educational curriculum that the study staff pediatricians taught to their doctor and nurse colleagues about the risks of hypothermia, how to provide optimal KMC, and how to use the Warmer when KMC is insufficient or unavailable. It included a hypothermia algorithm for sites with and without electric heat sources that is consistent with the Rwandan National Neonatal Protocol, but with the addition of the Warmer (Appendix 1). This thermoregulation training was followed by provision of 10 Warmers to each of 6 Rwandan district hospitals and 2 to each of the 84 associated health centers. Study sites were chosen because they each employed a Rwanda Paediatric Association (RPA) member pediatrician.

Our aim was to evaluate the impact of this intervention on the burden of neonatal hypothermia by comparing pre and post implementation data; assessing new benefits, barriers, and safety concerns regarding use of the Warmer outside of a strict research setting; and capturing the end-user’s experience with the Warmer.

Specifically, we compared 4 months of pre and post data consisting of de-identified information from hospital registries on admission temperature, hospital length of stay (LOS), and mortality rates. We also collected survey data from a Use Audit and a Non-Use Audit of HCPs interfacing with the Warmer. Finally, we conducted Likert scale and open-text pre and post interviews of HCPs and parents focused on perceptions and attitudes about thermoregulation. All of this data was collected from the hospitals only, not the health centers.

The training material recommended use of the Warmer for all newborns admitted to the neonatal ward of the participating hospitals if the newborns had a temperature <36.0 °C or were at risk of hypothermia due to a weight <2.5 kg when KMC was not available. There were no formal exclusion criteria except, per the thermoregulation protocol, patients who were unstable or <35 °C were to be given an electric heat source if available. The Warmer was also recommended for use in the Labor and Delivery wards. Newborns in associated health centers who were hypothermic were eligible to use the Warmer during stabilization and transfer to the district hospitals. While the intention was to use Warmers on transport to the district hospital with the goal of increasing admission temperatures, we did not collect transport data. Ultimate use of the Warmer was left to the discretion of the healthcare providers.

### Details of audits and surveys

**Warmer Use and Non-Use Audit:** The Warmer Use Audit provided data on the preparation, use, and cleaning of the Warmer, including any nonstandard practices or new safety concerns that might emerge when it was used outside of a research protocol. It included de-identified patient axillary temperature (obtained from the medical record) and ambient temperature (collected by study staff with a data storage thermometer) before and after Warmer use. The Warmer Non-Use Audit provided data on why the HCP chose not to use the Warmer despite a patient’s temperature being <36.0 °C. As their workflow and availability allowed over the 4 months after the Warmer was introduced, the pediatrician or a trained nurse at each hospital was to collect at least 8 Use Audits per month and up to 8 Non-Use Audits per month (depending on how many times it was noted that the Warmer was not used when indicated) at each site. This yielded a goal total of 192 Use Audits and up to 192 Non-Use Audits across all sites over 4 months.

**Qualitative interviews:** The pediatrician or trained nurse conducted Likert scale and qualitative interviews of HCPs and parents regarding the functionality of the Warmer; its interaction with KMC; and any perceived benefits, barriers, or safety concerns regarding its use. The Pre Surveys were designed to capture a wide range of opinions regarding the experience of HCPs and the well-being of the parents. The Post Surveys matched the Pre Surveys with additional questions regarding the strengths and weaknesses of the Warmer. The pediatrician or trained study nurse at each site conducted 6 Pre Surveys with HCPs and 6 with parents prior to introduction of the Warmers. As their workflow and availability permitted over the 4 months after the Warmer was introduced, the pediatrician or trained nurse at each site was to conduct 3 Post Surveys of HCPs and 3 of parents at each site per month. Thus our goal was to collect 36 Pre Surveys from HCPs and 36 from parents, and 72 Post Surveys from HCPs and 72 from parents, across all sites over 4 months. Based on our previous research, we exceeded the sample size needed to detect comparable results. Trained pediatricians or study nurses entered data into an electronic database, either directly or with the interim use of a provided paper form. There was no formal data validation process, but during data cleaning any incorrect data was excluded.

We employed standard methods of descriptive statistics (percentages, distributional parameters) to assess prevalent practices, problems, reasons for non-use, and users’ experience with and without the Warmer, including ease of use, attitudes, and impact on neonatal care. We applied standard inferential procedures (t-test and non-parametric analogues, contingency tables, factorial analysis of variance, multiple logistic regression) to make comparisons between phases where applicable. SAS software (Cary, NC) was used for all computations and, where hypotheses were to be tested, *p* <0.05 was taken as indicating statistical significance. Because the free-response answers were generally short and concrete, we grouped them into categories and analyzed them quantitatively. When there was more complex content, we included it as a direct quote.

**Ethical considerations:** The research was exempted by the Boston Children’s Hospital Institutional Review Board and the Rwandan National Ethics Committee on the basis of being a post surveillance study. Because the Warmer had already been studied extensively and raised no safety concerns with over 1000 uses while also demonstrating excellent efficacy in preventing and treating neonatal hypothermia, patients were not formally enrolled in the study and we did not ask for parental consent to use the Warmer. We did not obtain consent to collect de-identified data. Consent to complete the interviews was implied by participation. The identities of the HCPs and parents who participated in the surveys and interviews were not recorded.

## Results

We exceeded our goal for all audits and surveys except the Non-Use Audits at 188 out of 192 (Table 1). Since HCPs were instructed to complete this when they did not use the Warmer despite its being indicated, the number of these audits could not be prescribed. Many of the surveys were incomplete; our reported results are based on completed responses to each question.

**Table 1:** Number of Responses to Surveys and Audits.

SURVEY / AUDIT	NUMBER OF RESPONSES
Hospital Admission Data Pre Warmer	2398
Hospital Admission Data Post Warmer	1294
Warmer Use Audit	281
Warmer Non-Use Audit	188
HCP Pre Warmer Survey	49
HCP Post Warmer Survey	90
Parent Pre Warmer Survey	49
Parent Post Warmer Survey	97

Due to study staff turnover, we had pre Warmer data from 6 sites (totaling 2398 patients) and post Warmer data from 3 sites (totaling 1294 patients) (Table 2). Comparing sites with both pre and post data, the mean admission temperature rose from slightly hypothermic (36.41 °C) pre intervention to euthermic (36.53 °C) post intervention (*p* = 0.002). Two sites’ mean pre Warmer admission temperature was already euthermic (36.74 °C at each site), and neither their admission temperature nor percent euthermia on admission changed. A third site had a mean pre Warmer admission temperature of 35.76 °C that rose to 36.0 °C post Warmer (*p* = 0.0005) and a percent euthermia on admission of 27% that rose to 34% post Warmer (*p* = 0.02). There was no change in LOS or mortality in the pre compared to post data (Table 3).

**Table 2:** Hospital Admission Data Pre and Post Warmer.

HOSPITAL DATA PRE AND POST WARMER						
**Outcome**	**Hospital**	***n*: 2398**	***n*: 1294**			
		**Pre Warmer**	**Post Warmer**	**Pre Warmer**	**Post Warmer**	
				%	%	p*
Hypothermic	All sites			34.6	34.5	—
Euthermic	All sites			57	55.7	—
Hyperthermic	All sites			8.4	9.8	—
				Mean ± SE	Mean ± SE	p*
Admission temperature C	Sites w/ pre + post data	3	3	36.41 ± 0.03	36.53 ± 0.03	0.002
	All sites	6	3	36.46 ± 0.02	36.53 ± 0.03	—
	Hospital 1	526	375	36.74 ± 0.04	36.84 ± 0.05	0.14
	Hospital 2	446	491	36.74 ± 0.05	36.76 ± 0.05	0.75
	Hospital 3	428	423	35.76 ± 0.05	36.00 ± 0.05	0.0005
	Hospital 4	426	—	36.38 ± 0.05	—	—
	Hospital 5	302	—	36.68 ± 0.06	—	—
	Hospital 6	252	—	36.45 ± 0.06	—	—
				% ± SE	% ± SE	p*
Euthermia at admission†	Hospital 1	526	375	70 ± 2	70 ± 2	0.88
	Hospital 2	446	491	63 ± 2	63 ± 2	0.97
	Hospital 3	428	423	27 ± 2	34 ± 2	0.02
	Hospital 4	426	—	66 ± 2	—	—
	Hospital 5	302	—	69 ± 3	—	—
	Hospital 6	252	—	40 ± 3	—	—
				Median (min−max)	Median (min−max)	p*
Length of stay	Hospital 1	526	374	5 (0−69)	6 (0−64)	0.77
	Hospital 2	451	494	5 (1−39)	5 (0−68)	0.63
	Hospital 3	412	402	6 (0−60)	6 (0−77)	0.46
	Hospital 4	426	—	4 (0−60)	—	—
	Hospital 5	302	—	5 (1−51)	—	—
	Hospital 6	252	—	4 (0−39)	—	—
				% ± SE	% ± SE	p*
In-hospital mortality	Hospital 1	526	375	10 ± 1	7 ± 1	0.19
	Hospital 2	451	494	2 ± 1	4 ± 1	0.2
	Hospital 3	433	404	10 ± 1	12 ± 2	0.31
	Hospital 4	426	—	8 ± 1	—	—
	Hospital 5	302	—	9 ± 2	—	—
	Hospital 6	252	—	13 ± 2	—	—

*From factorial analysis of variance (temperature, log-transformed length of stay) or multiple logistic regression (euthermia, mortality), stratified with interaction terms to allow for variability among hospitals.

†Euthermia: 36.5−37.5 ºC.

**Table 3:** Warmer Use Audit.

WARMER USE AUDIT	*N*: 281
Pre-existing safety concerns	*n*: 266
Yes (milial rash)	2 (1%)
No	264 (99%)
	
Ongoing safety concerns during/after Warmer use	*n*: 270
Yes (milial rash)	1 (1%)
No	269 (99%)
	
Mattress placed in thermos prior to adding boiled water	*n*: 281
Yes	278 (99%)
No	3 (1%)
	
Mattress prepared in advance of need	*n*: 280
Yes	263 (94%)
No	17 (6%)
	
Mean time to prepare the Warmer when not ready in advance (minutes)	
	31 minutes | SD: 10 minutes, range: 5–60
	
Temperature indicator used correctly	*n*: 278
Yes	273 (98%)
No	5 (2%)
	
Deviation from normal use	*n*: 270
Yes (blanket placed between infant and Warmer)	2 (1%)
No	268 (99%)
	
Body temperature (°C) at start of Warmer use	
	mean: 35.9 °C, SD: 0.8°C, range: 32.0 °C–37.2 °C
	
Maximum body temperature (°C) during Warmer use	
	mean: 36.7 °C, SD: 0.5°C, range: 33.4° C–37.9 ° C
	
Duration of Warmer use	
	mean: 2h 03m, SD: 1h 16 m, range: 02m–6h 0m
Reasons for Warmer discontinuation	*n*: 276
Mother requested to resume KMC	178 (64%)
Warmer became cold	43 (16%)
Other	55 (20%)
Electric heat source became available	24 (9%)
Warmer used for transport then infant was admitted	23 (8%)
Infant was not warming	5 (2%)
Infant reached euthermia	3 (1%)
	
Warmer cleaned correctly	*n*: 260
Yes	251 (97%)
No	9 (3%)
	
Problems	*n*: 202
None	142 (70%)
Warmer not available when needed (not prepared in advance)	17 (8%)
Water scarce for use to heat mattress	10 (5%)
Necessary supplies to clean Warmer scarce	9 (4%)
Component of Warmer shows signs of breakdown (leakage…)	2 (1%)
Other	21 (11%)
Not enough kettles	7 (3%)
Lack of training	6 (3%)
Not enough Warmers	4 (2%)
Warmer prep time (too long)	4 (2%)
Baby not warming	1 (1%)

**Figure 4:**
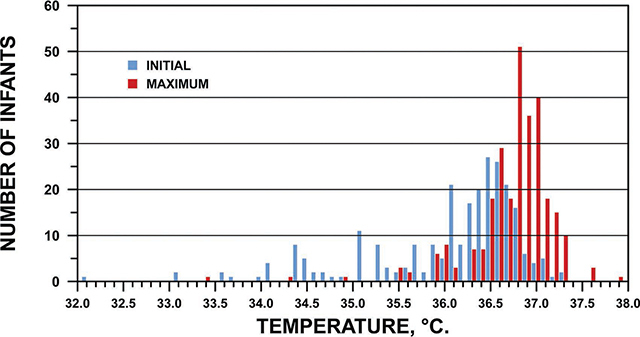
Initial and Maximum Temperature on Warmer.

There were a total of 281 uses of the Warmer, each described with a Warmer Use Audit. No new safety concerns were raised; 99% reported no concerns. Two nurses noted a milial rash before their patients were put on the Warmer, one of which resolved while on the Warmer. The Warmer was prepared, used, and cleaned correctly in the vast majority of cases. Specifically, the mattress was placed in the thermos before the boiled water was poured in (to avoid contact with the water) in 99% of audits. The mattress was prepared in advance of need in 94% of audits. The mean time to prepare the Warmer was 31 minutes with a range of 5–60 minutes (explained due to the nurse needing to attend to other tasks). The temperature indicator was used correctly in 98% of audits. The Warmer was used correctly in 99%, with 2 instances when a blanket was placed between the patient and the Warmer (reducing heat transfer). The mean temperature of the newborn on initiation of the Warmer was 36.0 °C with a range of 32.0–37.2 °C (Figure 4). The maximum temperature of the patient on the Warmer was a mean of 36.7 °C with a range of 33.4–37.9 °C*.* A temperature of ≥36.5 °C was achieved in 86% of audits. Due to missing data, the rate of temperature increase was discernible in only 32 of the 39 cases in which the temperature did *not* rise to ≥36.5 °C. Of these, 72% were warming at a goal rate of ≥0.5°C/hour (data not shown). In only 1% of audits were patients hyperthermic, with temperatures ranging from 37.6 to 37.9 °C. The mean duration of use was 2 hours 3 minutes with a range from 2 minutes to 6 hours. The reason for discontinuation of the Warmer included the pre-populated option that the mother requested to resume KMC (64%), the Warmer had cooled (16%), and “Other” (20%). It was cleaned correctly in 97% of audits.

When asked to describe any problems with the Warmer, 70% of respondents reported none, with some offering positive comments. The remainder chose from 5 pre-populated responses: Warmer not available when needed (8%), water [needed to heat Warmer] too scarce (5%), cleaning supplies scarce (4%), Warmer showed signs of breakdown (1%), and “Other” (11%).

The 188 Non-Use Audits (Table 4) provided 10 pre-populated reasons for not using the Warmer when indicated; 216 reasons were cited because respondents could choose more than 1 answer. Of these, the most frequently chosen was the Warmer was not prepared in advance of need (28%). The next 2 most common answers both pertained to inadequate time “to use the Warmer” (17%) and “to boil the water” (17%). Another 16% did not remember to use the Warmer, and 11% cited that there was no Warmer available as all were in use. The remaining choices were selected infrequently, with one instance in which an HCP had concerns about the safety of the Warmer, and one in which a family member had concerns about the Warmer, with no further details provided. The open-text comments largely reiterated and/or added details to the preselected answers, but some introduced new issues (data not shown). Specifically, 9% raised the problem of inadequate knowledge of hypothermia or training on use of the Warmer: “Some staff need to…know hypothermia is dangerous.” Related to training, 6% were unsure of the safety of the Warmer when the baby was unstable (2%), small (2%), or had birth asphyxia (2%). Another 2% raised concerns regarding unavailability of electricity to boil water. One nurse stated that she did not use the Warmer because of concerns regarding a skin rash.

**Table 4:** Warmer Non-Use Audit.

WARMER NON-USE AUDIT	*N*: 216*
Warmer not prepared in advance	61 (28%)
Did not have time to use	37 (17%)
Insufficient time to boil water	36 (17%)
Forgot to use Warmer	35 (16%)
All Warmers were already in use	24 (11%)
Absence of electricity	9 (4%)
Warmer efficacy concerns	9 (4%)
Water too scarce	3 (1%)
Family member declined use	1 (1%)
Safety concerns	1 (1%)

* 188 respondents provided 216 reasons for Non-Use because they could choose more than 1 answer.

HCPs filled out 49 Pre and 90 Post Surveys respectively (Table 5a), with a general trend toward improvement in a variety of attitudes toward thermoregulation and related topics. Hypothermia was correctly defined in 73% of Pre and 68% of Post Surveys. All HCPs thought it was important to keep babies warm both Pre and Post, and 88% Pre and 100% Post felt confident in doing so using KMC; 96% Pre and 100% Post liked helping mothers provide KMC; and 98% Pre and 100% Post felt confident taking care of small and sick babies. Only 57% Pre but 100% Post thought babies gained weight quickly while in the hospital.

**Table 5a:** Health Care Provider Survey (Pre and Post).

HCP PRE AND POST WARMER SURVEY		
	**Pre Warmer – *n*: 49 (100%)**	**Post Warmer – *n*: 90 (100%)**
What is the lowest normal temperature for a baby?	*n*: 48	*n*: 22
Correct	35 (73%)	15 (68%)
< 36.5	35 (73%)	15 (68%)
Incorrect	13 (27%)	7 (32%)
< 35.5	3 (6%)	4 (18%)
<34.5	5 (10%)	1 (5%)
other	5 (10%)	2 (10%)
		
Importance for keeping baby at a normal temperature	*n*: 49	*n*: 23
Very important	44 (90%)	21 (91%)
Important	5 (10%)	2 (9%)
Neutral	0	0
Unimportant	0	0
Very unimportant	0	0
		
Confidence of using KMC to keep baby warm	*n*: 49	*n*: 23
Very confident	17 (35%)	16 (70%)
Confident	26 (53%)	7 (30%)
Neutral	4 (8%)	0
Unconfident	2 (4%)	0
Very unconfident	0	0
		
Feelings about helping mothers provide KMC	*n*:49	*n*: 23
Like it a lot	43 (88%)	23 (100%)
Like it a little	4 (8%)	0
Neutral	2 (4%)	0
Dislike it a little	0	0
Dislike it a lot	0	0
		
Overall feelings about taking care of a small/sick baby	*n*: 49	*n*: 23
Very confident	26 (53%)	16 (70%)
Confident	22 (45%)	7 (30%)
Neutral	1 (2%)	0
Unconfident	0	0
Very unconfident	0	0
		
Rate in which small/sick babies gain weight in hospital/health center	*n*: 49	*n*: 23
Very fast	10 (20%)	8 (35%)
Fast	18 (37%)	15 (65%)
Neutral	7 (14%)	0
Slow	12 (24%)	0
Very slow	2 (4%)	0

In the HCP Post survey (Table 5b), the majority reported it to be easy to learn and remember to use (96%), prepare (92%), prepare in advance of need (85%), use the temperature indicator (91%), and clean (92%). Seventy-two percent reported it facilitated performing other required duties. All HCPs found it performed effectively to keep babies warm. Of respondents, 4% reported that it was uncomfortable for mothers to use in addition to KMC, and 43% noted that they had used it instead of KMC when KMC was indicated according to the thermoregulation algorithm. The Warmer was reported to promote KMC (79%), breastfeeding (89%), maternal bonding with their baby (81%), maternal well-being (86%), maternal physical recovery (94%), and mothers’ sense that their babies would survive and thrive (88%). All HCPs thought the Warmer looked both safe and well built. Ninety percent thought it worked well in their practice setting, with concerns related primarily to the need for more training.

**Table 5b:** Additional Health Care Provider Post Warmer Survey Questions.

HCP POST WARMER SURVEY (ADDITIONAL QUESTIONS)	*N*: 90				
	Very easy	Easy	Neutral	Difficult	Very difficult
Learning and remembering how to use the Warmer	45 (51%)	40 (45%)	2 (2%)	2 (2%)	0
Preparing the Warmer	47 (53%)	35 (39%)	2 (2%)	5 (6%)	0
Prepare the Warmer in advance	24 (32%)	40 (53%)	4 (5%)	7 (9%)	0
Using the temp indicator to determine if safe for use	47 (53%)	34 (38%)	7 (8%)	1 (1%)	0
Cleaning the Warmer	41 (46%)	41 (46%)	7 (8%)	1 (1%)	0
Warmer’s affect on ability to perform other required duties during shifts	22 (24%)	43 (48%)	14 (16%)	11 (12%)	0
					
	Very effective	Effective	Neutral	Ineffective	Very Ineffective
Ability of Warmer to keep baby warm	43 (48%)	46 (52%)	0	0	0
					
	Very comfortable	Comfortable	Neutral	Uncomfortable	Very Uncomfortable
Comfort of mother using Warmer while providing KMC	21 (23%)	52 (58%)	13 (14%)	3 (3%)	1 (1%)
					
	Very often	Often	Neutral	Almost never	Never
Warmer used instead of KMC when thermoregulation algorithm indicated to use KMC	12 (16%)	21 (27%)	12 (16%)	7 (9%)	25 (32%)
					
	Strongly promotes	Promotes	Neutral	Discourages	Strongly Discourages
Warmer effect on:					
breastfeeding	24 (31%)	45 (58%)	4 (5%)	5 (6%)	0
ability to provide KMC	23 (29%)	39 (50%)	9 (12%)	7 (9%)	0
mother/infant bonding	23 (29%)	41 (52%)	9 (11%)	6 (8%)	0
mother’s sense of well-being	22 (28%)	45 (58%)	10 (13%)	1 (1%)	0
mother’s physical recovery after childbirth	21 (27%)	53 (67%)	5 (6%)	0	0
Mother’s confidence baby will survive and thrive	22 (25%)	56 (63%)	9 (10%)	1 (1%)	1 (1%)
					
	Yes	No			
Does the Warmer look safe	81 (100%)	0			
Does the Warmer look well built	82 (100%)	0			
Concerns about Warmer working in your setting	3 (9%)	27 (90%)			

The most common responses regarding what they liked most about the Warmer (Table 5c) were that it worked well to provide additional heat (63%) and was easy to use (18%). What they liked least was the preparation time (39%), with 32% citing no dislikes. When asked what they would change about the Warmer, 34% of responses were related to the design (shorter, and unspecified change in length, shape, color, and weight), 14% would decrease the time it takes to prepare, 5% wanted it to last longer, while 42% wanted no changes.

**Table 5c:** Healthcare Provider Post Warmer Survey – Free-Response Answers.

CATEGORY + ANSWER	*N*: 199 (100%)
What did you like?	*n*: 71
Effectively warms/prevents hypothermia	45 (63%)
Easy to use	13 (18%)
Design (shape, size, material, portability)	10 (14%)
Other	3 (4%)
comfortable with KMC	1
safe to use in transit from community hospitals	1
provides heat while mother recovers	1
	
What did you dislike?	*n*: 69
Preparation (time)	27 (39%)
Difficult to use	6 (9%)
Design (shape, size, color)	3 (4%)
Uncomfortable	2 (3%)
Other	9 (13%)
make electric	1
not functioning properly (leakage)	1
concerned about water usage	3
need more training	1
cleaning	2
transportation is not easy	1
No dislikes	22 (32%)
	
What would you change?	*n*: 59
Design	20 (34%)
length	12
shorter	2
unspecified	10
shape	1
color	6
weight	1
Duration of warmth	3 (5%)
More training	2 (3%)
Decrease prep time	8 (14%)
Other	3 (5%)
increase quantity of Warmers	2
make it single use	1
No changes	23 (42%)

All of the Parent Surveys were completed by mothers: 49 Pre and 97 Post (Table 6a). Their pre and post intervention attitudes toward thermoregulation and KMC were positive. Both groups thought it was important that their baby have a normal temperature (100% Pre vs 94% Post). In both surveys, a minority were *not* providing KMC (2% Pre vs 11% Post). Both Pre and Post all liked providing KMC, felt confident keeping their babies warm with KMC (96% Pre vs 95% Post), felt their babies were gaining weight well (94% Pre vs 84% Post), were confident that they could provide sufficient milk (82% Pre vs 81% Post), and were generally confident being the parent of a newborn baby (94% Pre vs 90% Post). Only 49% Pre vs 48% Post felt physically rested.

**Table 6a:** Parent Survey (Pre and Post).

PARENT PRE AND POST WARMER SURVEY		
	**Pre Warmer – *n*: 49 (100%)**	**Post Warmer – *n*: 97 (100%)**
Is it important to keep your baby’s temperature in a normal range?	*n*: 48	*n*: 31
Yes	48 (100%)	29 (94%)
No	0	0
I don’t know	0	2 (6%)
		
Have you been providing KMC?*	*n*: 49	*n*: 97
Yes	48 (98%)	20 (21%)
No	1 (2%)	11 (11%)
Missing	0	66 (68%)
		
How much do you like providing KMC for your baby?	*n*: 47	*n*: 20
Very much	32 (68%)	15 (75%)
Much	15 (32%)	5 (25%)
Neutral	0	0
Little	0	0
Very little	0	0
		
Do you feel confident providing warmth through KMC?	*n*: 48	*n*: 20
Very confident	29 (60%)	12 (60%)
Confident	17 (35%)	7 (35%)
Neutral	2 (4%)	1 (5%)
Unconfident	0	0
Very unconfident	0	0
		
How well do you think your baby is gaining weight in the hospital?	*n*: 49	*n*: 31
Very well	21 (43%)	12 (38%)
Well	25 (51%)	14 (45%)
Neutral	3 (6%)	4 (13%)
Poorly	0	1 (3%)
Very poorly	0	0
		
Do you feel confident in having an adequate milk supply?	*n*: 49	*n*: 31
Very confident	18 (37%)	7 (23%)
Confident	22 (45%)	18 (58%)
Neutral	8 (16%)	1 (3%)
Unconfident	1 (2%)	4 (13%)
Very unconfident	0	1 (3%)
		
Overall how do you feel as the parent of your newborn baby?	*n*: 49	*n*: 31
Very confident	32 (65%)	15 (48%)
Confident	14 (29%)	13 (42%)
Neutral	2 (4%)	1 (3%)
Unconfident	1 (2%)	2 (6%)
Very unconfident	0	0
		
How have you felt physically since your baby was born?	*n*: 49	*n*: 31
Very rested	6 (12%)	1 (3%)
Rested	18 (37%)	14 (45%)
Neutral	10 (20%)	7 (23%)
Tired	14 (29%)	5 (16%)
Very tired	1 (2%)	4 (13%)

* Due to the large percentage of missing data, we calculated parents reporting that they did not perform KMC in the numerator, and total responses in the denominator.

In the Parent Post Survey (Table 6b), mothers overwhelmingly reported that the Warmer kept their baby warm (92%) and promoted KMC (73%), breastfeeding (75%), bonding (73%), their well-being (82%), their physical recovery (91%), and their confidence that their babies would survive and thrive (87%). While one mother wrote, “I think it can burn my baby,” other mothers overwhelmingly reported that the Warmer looked safe (99%) and well built (100%).

**Table 6b:** Additional Parent Post Warmer Survey Questions.

PARENT POST WARMER SURVEY	*N*: 97				
	Very well	Well	Neutral	Poorly	Very poorly
How did the Warmer work to keep your baby warm? (*n*: 97)	50 (52%)	39 (40%)	4 (4%)	4 (4%)	0
					
	Strongly promotes	Promotes	Neutral	Discourages	Strongly Discourages
What was the Warmer’s effect on:					
your ability to provide KMC (*n*: 96)	39 (41%)	31 (32%)	22 (23%)	4 (4%)	0
your ability breastfeeding (*n*: 96)	34 (35%)	38 (40%)	20 (21%)	4 (4%)	0
bonding with your baby (*n*: 97)	43 (44%)	28 (29%)	19 (20%)	7 (7%)	0
your sense of well-being (*n*: 97)	40 (41%)	40 (41%)	16 (16%)	1 (1%)	0
your physical recovery after childbirth (*n*: 87)	50 (57%)	29 (33%)	8 (9%)	0	0
your confidence that your baby will survive and thrive (*n*: 84)	52 (62%)	21 (25%)	11 (13%)	0	0
					
	Yes	No			
Does the warmer look safe? (*n*: 81)	80 (99%)	1 (1%)			
Does the warmer look well built? (*n*: 81)	81 (100%)	0			

When asked what they liked most about the Warmer (Table 6c), mothers responded with themes related to provision of warmth (+/− without need for incubator) (42%), ease of use (27%), and comfort (22%). Single comments included “I get some few hours of sleep in my favorite position as the baby is on Dream Warmer”; “It provides the heat to my baby when I am going to eat”; “It doesn’t take away baby from mother”; and “It promotes KMC.”

**Table 6c:** Post Warmer Parent Survey – Free Response.

CATEGORY + ANSWER	*N*: 189 (100%)
What did you like?	*n*: 67
Effectively warms/prevents hypothermia	32 (42%)
Easy to use	18 (27%)
Comfortable	6 (22%)
Other	11 (16%)
Helps mother:	3
Provide KMC	1
Bond with baby	1
Recover after childbirth	1
Portable	7
Shape and size	1
	
What did you dislike?	*n*: 64
Prep time/ease of use	17 (27%)
Heavy	13 (20%)
Uncomfortable	5 (8%)
Hard when cold	3 (5%)
Other	5 (8%)
Size	2
Doesn’t support KMC	1
Make it electric	1
Separates me from my baby	1
No dislikes	21 (33%)
	
What would you change?	*n*: 58
Design	26 (45%)
Color	9
Smoother	5
Smaller	4
Resize (unspecified)	4
Longer	3
Lighter	1
Other	12 (21%)
More Warmers in hospital	3
Be able to fully wrap the baby	2
More comfortable for KMC	1
Softer to allow breastfeeding while baby is on Warmer	1
Make a part that covers the head	1
Easier to prepare	4
No changes	20 (34%)

What they liked least was that it takes long to prepare (27%) and that it is heavy (20%), uncomfortable (5%), and hard when cold (5%). Despite the query, 33% reported no problems or positive attributes. Their recommendations to change the Warmer were primarily related to its design (45%), requesting that it be a different color; smoother, smaller, longer, lighter; or generally resized, with 34% wanting no changes.

## Discussion

We sought to evaluate the impact of a specific heat-producing wrap called the Dream Warmer on clinical outcomes related to neonatal hypothermia, and to broadly characterize the individual and systemic factors affecting its adoption and use under real-world conditions. We provided training and Warmers to 6 of the 42 district hospitals and 84 of the 499 health centers, thus involving approximately 15% of the relevant Rwandan healthcare facilities [10].

The pre and post Warmer data overall demonstrated a lower-than-expected rate of admission hypothermia at ~34%, well below the 64% recently reported even in middle-income countries [11]. This data also showed improvement in admission temperature after our intervention, largely driven by the hospital with a hypothermic mean admission temperature. Improving admission temperature reflects use of the Warmer prior to admission (e.g., during inter- and intra-facility transport). The lack of change in hospital LOS and mortality rates may reflect a “dose response” effect with need for the Warmer to be used more frequently throughout the hospitalization. This finding underscores the need to educate and support hospital staff to provide a continuous external heat source in order to improve these outcome measures.

The Warmer Use Audits confirm the safety of our three clinical trials, with no new concerns except the mention of a common, transient newborn rash seen for two patients prior to placement on the Warmer. With regard to efficacy, 86% of patients became warm, comparing favorably to the 92% in the composite data of our clinical trials, and 89% in a similar implementation science study in Malawi [12]. The rate of hyperthermia was only 1%, lower than our composite rate of 9% in our clinical trials, 10% in Malawi, and 12% in control patients *not* using the Warmer [9]. It was generally prepared, used, and cleaned appropriately but elicited some questions about the thermal indicator and some workflow issues pertaining to advanced preparation and cleaning. The finding that mothers requested to resume KMC well before the Warmer had lost its heat suggests that mothers are not seeking to maximize their time away from KMC.

The Non-Use Audits yielded valuable information about why HCPs did not use the Warmer when clinically indicated. Their lack of time factored into the most common challenges associated with use of the Warmer. The Warmer not being prepared in advance of need, and inadequate time to use the Warmer or boil the water are all facets of the core problem of the overtasked HCPs [13]. Though it would entail a change in workflow habits, preparation of the Warmer in advance of need would solve 62% of the reasons cited for non-use (e.g., Warmer not prepared, inadequate time to use Warmer and to boil water). It takes only a few minutes of hands-on time to prepare the Warmer, and once warmed in the thermos, it remains ready to use for approximately six hours; yet, this does require anticipation of need. The concern regarding use of the Warmer with newborns who are unstable, small, or have birth asphyxia reflects an educational gap. The Warmer was specifically designed to enable ready access to patients for both assessments and procedures, to accommodate any sized patient, and to stabilize the temperature of newborns with birth asphyxia in order to avoid the marked hypo- and hyperthermia that can compound their neurologic injury [14].

The HCP Pre and Post Surveys demonstrated a good baseline knowledge that generally improved after the intervention (training and provision of the Warmers). The Post Surveys overall demonstrated that the Warmer was easy to use and positively promotes other aspects of the HCPs’ and mothers’ experience. The only concern is that 43% of HCPs responded that they had used the Warmers instead of KMC, with no further information provided. The Warmer is intended to supply external heat when a mother is not available for KMC, but it is critical that the Warmer does not detract from or compete with KMC. That 79% of HCPs report the Warmer promotes KMC is reassuring.

The Parent Pre vs Post Surveys generally reveal a positive understanding of the importance of euthermia and mothers’ role in providing KMC. One concern is the rise in reported rates of not providing KMC from 2% Pre to 11% Post. Without knowing true rates of KMC before and after, it is difficult to know the significance of this finding. It is reassuring that 73% of parents report that the Warmer promotes KMC. Again, any evidence that the Warmer detracts from KMC must be noted and addressed. The other valuable lesson from the Parent Post Survey is that some mothers find the Warmer heavy to use in addition to KMC. The 1 kg weight of the Warmer was chosen so that it will stay warm for approximately 6 hours. The mass of the wax relates directly to the duration of heat provision. Since the majority of mothers requested no change in design, and several HCPs requested that the Warmer provide heat for a *longer* duration, it would be helpful to observe how mothers position the Warmer to learn if there are techniques that optimize comfort rather than decrease the mass of wax.

As might be expected, the HCPs commented more on issues related to usability, while the parents were more concerned with the comfort and weight of the Warmer. Across the HCP and Parent Post Surveys, the likes, dislikes, and requested changes mostly balanced each other out in terms of wanting it shorter, longer, lighter, heavier, and so on. The exceptions were two recurring issues that spanned all the audits and surveys that must be robustly addressed for the Warmer to be optimally and sustainably integrated into thermoregulatory care:

- lack of education and training regarding the importance of euthermia and instructions for use of the Warmer
- lack of time to prepare, use, and clean the Warmer

Regarding the perennial problem of education and training when introducing a new practice [15], we are currently making three open-access videos. One will be for HCPs and a second for family members in which we will provide education on the risks of hypothermia, the importance of a continuous external heat source to provide a thermal-neutral environment, how to provide high-quality KMC, and use of a heat-producing wrap when KMC is insufficient or unavailable. A third video will demonstrate the preparation, use, and cleaning of the Warmer.

To address the common problem of the overstretched HCP, we are beginning to study the potential role of family-centered care (FCC) [16,17] as a novel approach to improve thermoregulatory infant care. This lends itself well to FCC because most of the diagnostic and therapeutic procedures can be feasibly performed by family members: taking a temperature, teaching high-quality KMC, and preparing a simple heat-producing wrap such as the Warmer when KMC is either insufficient or unavailable. By shifting the task of thermoregulation to parents, nurses could better attend to other critical portions of their jobs. Parents teaching KMC to other parents could also provide valuable peer-to-peer support. They will likely need to continue attending to thermoregulation in the outpatient setting, therefore building these skills while inpatient would both facilitate their discharge teaching and enhance parenting skills.

Our study has several limitations. As an implementation science study with limited funding and personnel, we did not have the resources to ensure all audits and surveys were complete, or to measure many variables of interest. This includes actual rates of KMC pre and post intervention. Because of this, we cannot make a definitive comment about the effect of the Warmer on KMC, whether positive, neutral, or negative. There are no validated surveys that we could use to address this highly specific topic, and therefore we cannot know if there were any gaps in respondents’ understanding of the questions. There was also a language barrier, with study personnel needing to translate the Parent Surveys from the Kinyarwanda language, potentially introducing error. Both HCP and Parent Surveys could have been influenced by a bias to please the research personnel, who could have been perceived as powerful hospital staff. Finally, the study was limited to rural health centers and district hospitals in Rwanda; the Warmer may be received differently in other settings, thus limiting the generalizability of our results.

## Conclusion

Heat-producing wraps are the WHO’s recommended method for improving thermal care when KMC is insufficient or unavailable. The Dream Warmer is an example of such a product that has performed well in formal clinical trials, and again in this implementation science study. We are producing educational videos to address the ongoing need for training, with a clarifying message that heat-producing wraps serve to complement KMC. Engaging families in thermoregulatory care could both offload the overburdened HCP and enhance parental skills. Given the morbidity and mortality toll of neonatal hypothermia, it is urgent that this preventable condition be minimized.
